# The Higher, More Complicated: The Neural Mechanism of Hierarchical Task Switching on Prefrontal Cortex

**DOI:** 10.3390/brainsci12050645

**Published:** 2022-05-14

**Authors:** Chengdong Zhu, Jiahui Han

**Affiliations:** 1 School of Physical Education, Liaoning Normal University, Dalian 116029, China; zhuchengdong104321@lnnu.edu.cn; 2 Brain and Cognitive Neuroscience Research Center, Liaoning Normal University, Dalian 116029, China; 3 Key Laboratory of Brain and Cognitive Neuroscience, Liaoning Province, Dalian 116029, China

**Keywords:** task switching, hierarchical structure, prefrontal cortex, cognitive control

## Abstract

Cognitive control is essential to daily life. Task switching is a classical paradigm used to study cognitive control. Previous researchers have studied the representation of different abstract hierarchical rules in the prefrontal cortex and explored the process mechanisms of task switching. However, the differences between the different hierarchical levels of task switching, especially the related neural mechanisms in the prefrontal cortex, are still unclear. This review focuses on and summarizes this issue. The present study suggests that the higher the hierarchical rule shifting or task switching, the more anterior the activation is on the prefrontal cortex. In addition, a high hierarchy of rules or tasks is more abstract, which leads to a larger switching cost.

## 1. Introduction

Cognitive control is an essential topic in the field of cognitive neuroscience, and one of its main paradigms is task switching. A large number of studies explored and revealed the cognitive neural mechanism of task switching or rule switching [1,2,3]. In classical task switching paradigms, participants are presented tasks with two or more rules and are required to flexibly establish decisions for the task between these rules. Some researchers found that individuals will respond longer and make more mistakes in a switching condition than in a repetition condition, which is called the “switching cost” [4,5].

Researchers have conducted systematic studies on the causes and brain mechanisms of switching costs. However, there are still unresolved problems related to task switching. In particular, the hierarchical process in task switching must be systematically illustrated. In this paper, we summarize and elaborate upon the structure and function of hierarchy associated with task switching. The hierarchical structure related to task switching mainly refers to information on the upper and lower branch levels of rule structure (or orders and tasks) [6,7]. The higher level aggregates more complex rules and contains more abstract rules. In contrast, the lower level aggregates fewer rules and detailed rules specifically. In addition, previous studies proposed that individuals were inclined to handle rules from top to bottom in hierarchical structures [8]. In this context, the present study focuses on the function of hierarchy in task switching (such as temporal precedence and process dominance) based on different hierarchical information. In this study, we first review the mechanism of abstraction rule processing at different levels (i.e., the function of the prefrontal cortex in cognitive processing with different hierarchical level structures). We then summarize the neural mechanisms of classical task switching processing. Based on this, we focus on the neural mechanism of task switching with different levels of abstraction rules and its process mechanism in the prefrontal cortex. Finally, we propose prospects for future research.

## 2. Hierarchy of Rule Representation in the Prefrontal Cortex

Hierarchy plays an important role in understanding the organizational structure and cognitive activities related to behavioral goals [9,10,11]. When the brain performs lower hierarchical activities, it needs guidance and representation from higher hierarchical information; that is, information in a higher hierarchy can guide task processing in a lower hierarchy [9,10,12,13,14]. Researchers believe that high-level information processing is a superior and abstract level of structure with earlier representation [15]. For example, if someone attempts to keep a pet, the process of selecting the type of pet (cat vs. dog) is more abstract, which is a high hierarchical level structure. Accordingly, if he or she selects a dog, then choosing the kind of dog (golden retriever vs. German shepherd) is relatively more specific, which is a low hierarchical level structure. Consequently, low-level information can be processed only when high-level structure information is processed, and the task is completed.

**Figure 1:**
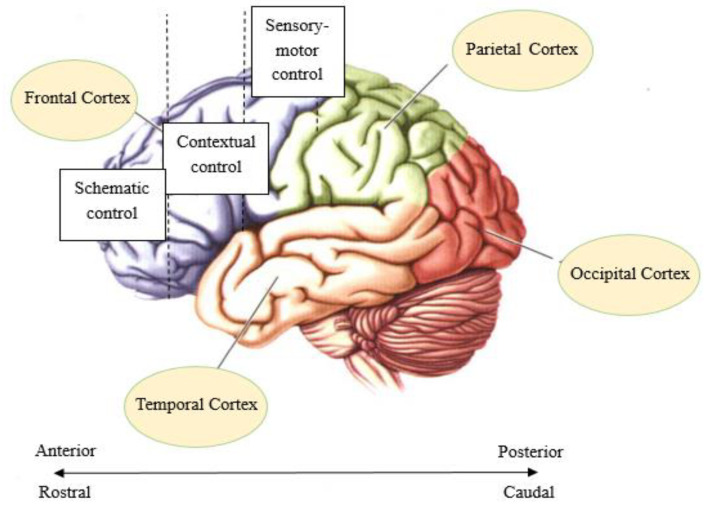
A hierarchical representation of cognitive control from the caudal to the rostral on the prefrontal cortex.

In recent years, some researchers have explored the relationship between the hierarchy of brain functions and the degree of abstraction of rule representation. For example, Badre and D’Esposito designed four tasks with different degrees of abstraction, and the degree of abstraction of rule representations increased successively [16]. The results show that the prefrontal cortex plays an important role in the processing of abstract representations. Importantly, there is a hierarchical organization of cognitive control from the caudal to the rostral on the prefrontal cortex (PFC) (Figure 1). When representing specific rules, the posterior prefrontal region is activated, and the anterior prefrontal region is activated when abstract rules are represented. This suggests that the more abstract the rule representation, the more anterior the activation region [6,7,16,17,18,19]. Similarly, the cascade model of cognitive control proposed by Koechlin et al. suggests that there is also a top-down executive control system in the lateral prefrontal cortex and premotor regions from the beak to the tail [20]. Specifically, episodic control, located at a high level, changes with the passing of episodic signals (past events) and is associated with selecting task sets. The low level of contextual control, which changes with the transmission of background signals, is related to the task setting itself. In addition, sensory control at a lower level changes with the information transmission of stimuli related to the response. The cascade model also showed that the individual responses were affected by these three hierarchical controls (sensory control, contextual control, and episodic control), and the response time was longer with increasing hierarchical control levels [20,21].

Although previous studies have explored the cognitive control system and the hierarchical characteristics of brain structure and function, as one of main contents in the field of cognitive control, the hierarchical process mechanism of task switching has not yet been clearly revealed. For this purpose, the main findings of task switching will be reviewed first, and then the recent research progress on hierarchical task switching will be demonstrated.

## 3. Cognitive Control and Task Switching

### 3.1. Switching Cost and Its Theoretical Explanations

Task switching is one of the main paradigms used in cognitive control studies. A large number of studies has found that individuals perform worse in the switching condition than in the repeat condition, called the phenomenon of switching cost [3,22,23,24,25,26,27]. There are two different theoretical explanations for the production of switching costs: the interference theory and reconfiguration theory. According to the interference theory, when individuals respond to the switching trial, switching costs are generated to overcome the interference caused by the activated task set from the last trial [28]. According to reconfiguration theory, the reason for switching costs is that individuals need to spend more time on endogenous and top-down control processing. For the new task to be transformed and to complete the task setting related to switching, executive control processing reconfigures the corresponding cognitive processing system. Owing to task reconfiguration, this additional control requirement produces switching costs [28,29,30]. In addition, some researchers have integrated these two theories, believing that both interference and reconfiguration play essential roles in task switching [1,22]. 

### 3.2. Prefrontal Cortex Related to Task Switching 

A large number of studies has found activation of the frontoparietal brain network during task switching [31,32]. Continuous activation of the right anterior prefrontal cortex was observed when comparing task switching and repetition blocks. The left superior parietal gyrus was significantly activated when the switching trials were compared to repeated trials [33].

Researchers have found that the specific brain regions responsible for processing vary according to the type of task switching [34]. During one classical type of task switching, both the parietal cortex [32] and the dorsal portion of the premotor cortex [34] play essential roles in perceptual switching (e.g., participants need to respond to a shape by pressing the left button for a circle or the right button for a square). In context switching (e.g., the Wisconsin card sorting task), both the frontopolar cortex [34] and the dorsolateral prefrontal cortex play dominant roles [32].

In addition, some studies explored the relationship between network connectivity and cognitive performance in relation to task switching. The results indicated that frontoparietal networks play an important role in task switching. For example, Vallesi et al. demonstrated that the frontoparietal network neural mechanism was involved during the different cue-to-target intervals (CTIs) of task switching [35]. Specifically, with lower homotopy in the superior frontal gyrus, the mixing costs of short CTI trials were lower. However, with lower homotopy in the supramarginal gyrus, the mixing costs of long CTI trials were lower. Furthermore, Tsumura et al. revealed distinctive neural mechanisms by comparisons across hemispheres [36]. Specifically, the participants were asked to decide the motion or color coherence of the stimulus based on the cue. The authors found that the repeat trials resulted in better performance (reaction time and accuracy) than the switch trials, and the higher-coherency trials performed better than the lower-coherency trials. Notably, the results demonstrated that task switching is related to the frontoparietal regions in the left hemisphere, whereas perceptual decision making of stimuli is associated with the frontoparietal regions in the right hemisphere. In addition, Uehara et al. elaborated on the left frontoparietal neural mechanism associated with task switching by utilizing two sequential finger-tapping tasks [37]. The results showed that the switched trials produced greater preparatory activity in the left frontoparietal cortices. However, when the performance of the switched trials improved, the left intraparietal activity decreased.

### 3.3. EEG Markers of Neural Mechanisms of Task Switching

Researchers have utilized numerous paradigms to study task switching, including voluntary and intermittent task switching. In particular, the target-cued task-switching paradigm (one of the classical task-switching paradigms) has been employed by a large number of studies investigating the mechanism between switching and repeated conditions [38,39,40,41,42]. Generally, two main stimuli where cues and targets emerged in succession were utilized in this paradigm. The cue stimuli with different perceptual features appeared initially, indicating the rules (e.g., parity vs. magnitude judgments) corresponding to the target. The target stimuli used were Arabic numerals (1–9, excluding 5), which were required to make decisions using keypresses. Accordingly, studies have compared and demonstrated differences in the neural mechanisms between switching and repeated conditions for the cue and target stimuli, respectively.

Recently, some researchers have used functional connectivity (FC) technology to investigate the brain mechanism of task switching, which is consistent with the results of functional imaging studies, and the frontoparietal brain network is closely related to task switching [1,28,38,39,43]. Several EEG studies applying the target-cued task switching paradigm have shown that cues and targets in task switching evoke different neural mechanisms which are related to cognitive control [44,45]. Differences in neural mechanisms can be observed in the theta band (4–8 Hz) [38,39,43,46]. In particular, cue-evoked θ oscillations are related to proactive control processing, such as information updates and expectations. Target-evoked θ oscillations are associated with reactive control processing, such as interference resolution [39,43]. Although both cue and target processing are accompanied by θ oscillations, the connection patterns of the brain networks are different [38,39]. Compared with the repeated condition, the cue stimuli in the switching condition were only associated with strong connections between most of the frontal cortex electrodes and a few parietal cortex electrodes. In contrast, the target stimuli in the switching condition had obvious brain network connections between a small number of frontal electrode sites and a large number of parietal electrode sites [39].

Other studies have shown that task switching processing is related to alpha activation (8–12 Hz) [46]. It was found that the cue stimulus in the switching condition increased the alpha activation in the frontoparietal cortex. The results suggested that individuals need to update rules or task sets, which promotes reactive control [40,47]. However, the target stimulus in the switching condition results in a decrease in alpha oscillation in the frontoparietal cortex [48], which is related to the process in executing the task [49]. 

## 4. Hierarchical Process Mechanisms of Task Switching

Individuals often perform multiple tasks in daily life and need to switch between them [1]. Tasks are usually organized in a hierarchy [50]. Accordingly, task switching would be affected by hierarchical representations [8,50,51]. 

### 4.1. The Generation Forms of Hierarchical Tasks

In some studies on task switching, hierarchical rules were constructed using experimental instructions. For example, in Schneider and Logan’s study [50], before the experiments, the participants were informed that in an ABAB task sequence, former groups AB and AB were regarded as two independent aggregate tasks. Nevertheless, the four trials in this sequence had to be evaluated [50]. In this case, the two aggregate tasks were high-level tasks, and trials A and B in each aggregation were regarded as task elements (i.e., low-level tasks). The results showed that the switching cost in high-level tasks was greater than that in low-level tasks [52]. Lien and Ruthruff explained that high-level tasks are more complex, and as the complexity of tasks increases, so does switching cost [53]. They also found that the switching effect in high-hierarchy tasks (ensemble level) dominated that in low-hierarchy tasks (element level). In other words, when the hierarchical structure is not established, the switching cost is mainly caused by the switching effect at the element level. However, when a hierarchical structure is established, the switching cost in ensemble-level tasks is higher than that in element-level tasks [53]. This is because the representations of task sequence and element task processing share the same working memory capacity, and sequence-level processing may change task-level processing [52].

In a hierarchical switching task, individuals can also spontaneously establish hierarchical structures and choose appropriate ways to transfer or reuse the information processed for whole hierarchical structures in a new environment [11]. For example, the context-task-set model (C-TS model) proposed by Collins and Frank describes how learners infer hidden hierarchical structures or rules and decide how to reuse the learned hierarchies in new situations or build new hierarchies using the learned information [9].

In the learning phase, the participants were presented with four stimuli with different colors and were required to make corresponding behavioral responses (A_1_ for a red triangle, A_2_ for a red circle, A_3_ for a yellow triangle, and A_4_ for a yellow circle). Similarly, in the test phase, the participants were still presented with two triangles and two circles with different colors, but the colors changed compared with the learning phase. Participants were asked to provide the following behavioral responses: A_1_ for the blue triangle, A_2_ for the blue circle, A_1_ for the green triangle, and A_4_ for the green circle. In the learning phase, the participants did not know that the color content was a potential high-level rule until they learned that. After learning, the participants spontaneously regarded color as a high-level rule. Specifically, in the testing phase, the shape stimuli were presented to the participants, which was the same as in the practice phase (triangle and circle). Although TS_4_ of the green shape stimulus was partly identical to the original TS_1_ and TS_2_, the participants realized that the task set of the blue shape stimulus was identical to the task set TS_1_ (A_1_ for the triangle and A_2_ for the circle) in the original C_1_ content (red). Consequently, by practicing in the learning phase, the participants will spontaneously use the color content as a higher-level rule in the testing phase.

### 4.2. Hierarchical Network Processing Models of Task Switching

The switching cost has an important effect on task switching. Collins and Frank [9] proposed a hierarchical network processing model related to switching costs in combination with the context-task-set model (C-TS model) introduced above [9]. Researchers assume that two neural loops exist in this model. The first loop is a task set loop, which is responsible for extracting and transferring high-level content (or rule) information (i.e., gathering content or rules with the same task set together). The second loop is the motor loop, which learns and transfers information about the motor response associated with choosing the task set and perceptual stimuli. The second loop can only be processed if the first loop is completed. Here, motor behavior is associated with the choice of the task set. If a conflict occurs in the selection of the task set, the reaction in the motor loop results in delayed processing.

In the experiment, the participants were asked to press Key 1 if the target was a yellow triangle; if it was a yellow circle, Key 2 was pressed; if it was a red triangle, Key 3 was pressed; and if it was a red square, Key 4 was pressed. Researchers have suggested that in the first loop, if participants spontaneously define the color as high-level content information, an association between color and the PFC will be formed first. Subsequently, a relevant task set was generated. The high-level rule (color: yellow and red) in the first loop is multiple. Therefore, if a wrong task set is chosen in the first loop, the frequency of wrong motor actions will be increased, and the switching cost for accuracy will be generated. If the neural network requires extra time to update the task set in the PFC and to overcome the wrong task set, it will generate a switching cost for the reaction time [9].

Accordingly, in the switching trial, if the color (high-level rule) changes, both loops are changed, and the overall task set needs to be updated. If the color does not change and only the shape (low-level rule) changes, the degree of task set updating is reduced. Consequently, the switching cost of high-hierarchy switching trials is larger than that of low-hierarchy switching trials [9].

### 4.3. Brain Mechanisms of Hierarchical Task Switching

Kleinsorge and Heuer [8] proposed a parameter model for a hierarchical structure for task switching. According to this model, if the high-level parameters change, all top-bottom-related hierarchical parameters are reconfigured. If the parameters at the lower level are changed, only the parameters at that level must be reconfigured. From this viewpoint, the switching cost depends on the number of changed parameters. Consequently, the hierarchical level at which the stimulus lies determines the switching cost (Kleinsorge and Heuer [8]. Accordingly, the switching cost of high-hierarchy rule shifting is greater than that of low-hierarchy rule shifting [51,54].

Few studies have revealed the brain mechanisms underlying hierarchical task switching. Collin et al. [12] showed that the participants could construct hierarchies of rules spontaneously, and the switching conditions evoked larger negative waves in the late time window (450–609 ms) [12]. Unfortunately, although significant differences were found between the switching and repeat conditions in the early time window, there was no statistical difference between the two hierarchical switching conditions (higher vs. lower levels) [12]. One possibility is that because of the reinforcement learning task, structure representation occurs with the task operation and cannot be observed explicitly.

Subsequently, Han et al. [51] modified Collins et al.’s paradigm [12]. To explore the processing mechanism of task switching between different hierarchical structures, they employed the classical target-cued task-switching paradigm. They explicitly presented the participants with different hierarchical stimulus structures [51]. The results showed that for cues, the difference between high hierarchical and low hierarchical rule shifting conditions appeared in the P2 time window in the frontal region, and the difference between the low hierarchical shifting and repeat conditions appeared in the N2 time window in the parietal region. However, the target-ERP pattern was opposite to the cue stimulus; that is, the difference between the repetition and shifting conditions first appeared in the P2 time window in the parietal region, while the difference between the high and low hierarchical shifting conditions appeared in the N3 time window in the frontal brain. Researchers believe that the cue phase is the process of rule learning in which a mastering rule structure is a prerequisite. Consequently, the hierarchical features of rules associated with the task stimulus would be processed first, in which the hierarchical effect (high and low shifting) appeared earlier. For the target, the task phase is the process of applying rules, in which the participants need to judge whether the rule of stimulus changes. If this is performed, individuals will further distinguish between high and low hierarchical shifting. Accordingly, the switching costs occur earlier than the hierarchical effect. Although Han et al. [51,54] demonstrated the processing mechanism of hierarchical rule shifting, more ERP research on hierarchical rule-shifting and task-switching and neural mechanisms needs to be explored and discussed in the future.

In addition, the neural oscillations of task switching with a hierarchical structure were investigated. The theta power would be increased, appearing in the rule (cue stimuli) at a high level, which reflected that individuals need more cognitive resources for the hierarchical control process. In addition, the alpha power will be decreased, accompanied by a high-level rule, which is related to the proactive control of rule updating [55]. Although some researchers have investigated hierarchical task switching on ERP components and oscillations, few studies have elaborated on the patterns of brain network connections. Since the prefrontal cortex plays an important role in hierarchical processing, the functional connection process of hierarchical task switching in the prefrontal cortex still needs to be illustrated.

## 5. Conclusions and Prospect

In task switching or rule shifting with a hierarchical structure, as the hierarchy control model proposed [19], a higher hierarchical level of control activated the anterior prefrontal region, whereas a lower hierarchical level of control activated the posterior prefrontal region [6,7,16,18]. Moreover, some researchers confirmed that frontoparietal networks have a close relationship with task switching [35,36,37]. Specifically, the process related to task switching is concentrated in the left frontoparietal region, whereas brain activation associated with perceptual decision making in response to stimuli takes place in the right frontoparietal network. Task switching is a subcomponent of cognitive control [35,56], and we speculated that compared with lower hierarchical task switching, the frontoparietal connectivity of higher hierarchical task switching was more intensive and anteriorly activated in the left hemisphere. However, there are still some problems to be solved.

First, although researchers have proposed the theory of hierarchical task switching and the hierarchical control model, few studies have directly explored the neural processing mechanisms of task switching or rule switching at different hierarchical levels. Specifically, when the brain represents abstract hierarchical tasks, brain regions are activated in the prefrontal cortex. The more abstract the rule representation, the more anterior the brain regions [6,7,17,57]. Although the prefrontal cortex is responsible for processing hierarchical switching tasks, the neural differences between high- and low-hierarchy switching tasks in the prefrontal cortex are unclear. We inferred that the higher the hierarchical rule shifting or task switching, the more anterior the activation of the prefrontal cortex. The question of the differences in processing mechanisms needs to be answered in future studies. In addition, although some studies have revealed the brain mechanism of task switching by utilizing the functional connectivity technology, the EEG connectivity pattern of hierarchical task switching is seldom investigated. Further research should explore this issue.

Second, cues and targets in task switching are associated with different brain patterns and cognitive mechanisms. Previous studies have also concluded that switching costs are closely related to executive control [1,28]. Although researchers believe that different stimuli (cue vs. target) involve different types of cognitive control (proactive control vs. reactive control) [39], processing of these two controls in rule shifting or task switching at different levels of abstraction has rarely been explored. Given that the activated patterns of these two controls are different in the frontoparietal network, what is the activated pattern of the brain network when cue stimuli are combined with target stimuli? As the hierarchy changes and the rule level increases, will the two control-processing resources be required more? Alternatively, will these two controls have a tradeoff? What is the relationship between these two controls and the prefrontal cortex? These questions require further investigation.

Finally, individuals know the hierarchical structures of rules, which can be either informed by instructions or created spontaneously by themselves. Although the prefrontal cortex is responsible for the representation of hierarchical rules with different levels of abstraction, what is the relationship between the acquisition of hierarchical structures and the brain-processing mechanism? What are the differences in the brain networks responsible for the processing? In addition, when the rules of a hierarchical structure are set up by individuals spontaneously, they need to practice first and then test them. With this in mind, what is the brain response difference between the practice and test phases when individuals process a hierarchical structure of rules? These problems need to be addressed in the future.
